# Molecular Characterization of Southern African Territories 2 (SAT2) Serotype of Foot-and-Mouth Disease Virus from Nigeria in 2017 to 2018

**DOI:** 10.1128/MRA.00362-21

**Published:** 2021-07-08

**Authors:** Bridget Fomenky, Kate Hole, Hussaini Ularamu, Yiltawe Wungak, David Ehizibolo, Michelle Nebroski, Peter Kruczkiewicz, Cody Buchanan, Oliver Lung, Charles Nfon

**Affiliations:** a National Centre for Foreign Animal Disease, Canadian Food Inspection Agency, Winnipeg, Manitoba, Canada; b National Veterinary Research Institute, Vom, Plateau State, Nigeria; KU Leuven

## Abstract

This report describes the nucleotide sequences of eight Southern African Territories 2 (SAT2) serotype foot-and-mouth disease virus strains from 2017 to 2018 outbreaks in cattle in Nigeria. These viruses belong to topotype VII of SAT2 and were closely related to previous isolates from Nigeria and other West African countries.

## ANNOUNCEMENT

Foot-and-mouth disease virus (FMDV), genus *Aphthovirus*, family *Picornaviridae*, causes a highly communicable disease of cloven-hoofed animals responsible for production losses and trade restrictions (1, 2). Seven serotypes (O, A, C, Asia 1, and South African Territories 1 [SAT1], 2, and 3) exist (3). Serotypes O, A, and SAT1, -2, and -3 have been reported in Nigeria (4–6). Due to constant evolution of FMDV and porous borders in West Africa, monitoring of circulating FMDV is required in order to facilitate vaccine selection.

Here, epithelial tissues in BD universal transport system vials (VWR, Canada) were collected from FMD outbreaks in Plateau State (PL) and Bauchi State (BAU) in Nigeria in 2017 to 2018 and sent to the National Centre for Foreign Animal Disease (NCFAD), Canada. Tissue homogenates (10%) were prepared in a Precellys tissue grinding kit (ESBE Scientific, Canada) and clarified (7). RNA was extracted using the MagMax viral RNA isolation kit (Life Technologies, Canada) (8) and tested for FMDV using real-time reverse transcription-PCR (rRT-PCR) as described previously (9). Near full-genome sequences of FMDV (Table 1) were obtained from rRT-PCR-positive samples using next-generation sequencing (NGS) (10). RNA for NGS was processed as described previously (11), including DNase treatment and RNA purification prior to first-strand cDNA synthesis. Illumina Nextera XT sequencing libraries were prepared according to the manufacturer’s instructions and sequenced on a MiSeq instrument using a V3 cycling kit (Illumina). Raw sequencing data were processed with the nf-villumina Nextflow (12) workflow (https://github.com/CFIA-NCFAD/nf-villumina) for quality control, taxonomic classification, and *de novo* assembly (13). *De novo* assembly was performed with Unicycler v0.4.7 (14) in “conservative” mode to find an optimal SPAdes assembly (15) for each set of paired-end Illumina reads. Contigs were identified as FMDV using nucleotide BLAST (16, 17) search against the NCBI nucleotide database. The gene segments, including VP1, were identified with Annotate in Geneious v9.1.8 using a publicly available annotated FMDV genome. Phylogenetic analysis of obtained and previously published VP1 sequences in GenBank was performed using the Molecular Evolutionary Genetics Analysis (MEGA X) software (18), with the evolutionary history inferred using the maximum likelihood method and Hasegawa-Kishino-Yano model. A discrete gamma distribution was used to model evolutionary rate differences among sites (5 categories [+G, parameter = 0.3453]). A bootstrap value of ≥70% was considered significant.

**TABLE 1:** Accession numbers and identification of the FMDV SAT2 viruses from Nigeria in 2017 to 2018

Sample name	Accession no.	Near-complete genome size (no. of nt)^*a*^	GC content (%)	Total reads	Avg read length prior to processing	Raw data identifier
SAT2/NIG/BAU/TR/1/2018	MW715622	7,638	53.6	479,752	139.0	SAMN19166821
SAT2/NIG/PL/JS/KA/1/2017	MW715623	7,625	53.8	665,056	101.1	SAMN19166820
SAT2/NIG/PL/KWK/02/2017	MW715624	7,657	53.7	373,482	137.9	SAMN19166819
SAT2/NIG/PL/LANG/02/2017	MW715625	7,649	53.7	424,730	138.5	SAMN19166818
SAT2/NIG/PL/PKN/01/2017	MW715626	7,673	53.6	366,152	139.7	SAMN19166817
SAT2/NIG/PL/PKN/02/2017	MW715627	7,698	53.0	408,684	141.0	SAMN19166816
SAT2/NIG/PL/WAS/01/2017	MW715628	7,330	53.9	481,220	137.2	SAMN19165925
SAT2/NIG/PL/WAS/03/2017	MW715629	7,601	53.8	404,476	140.2	SAMN19165530

a nt, nucleotides.

**FIG 1:**
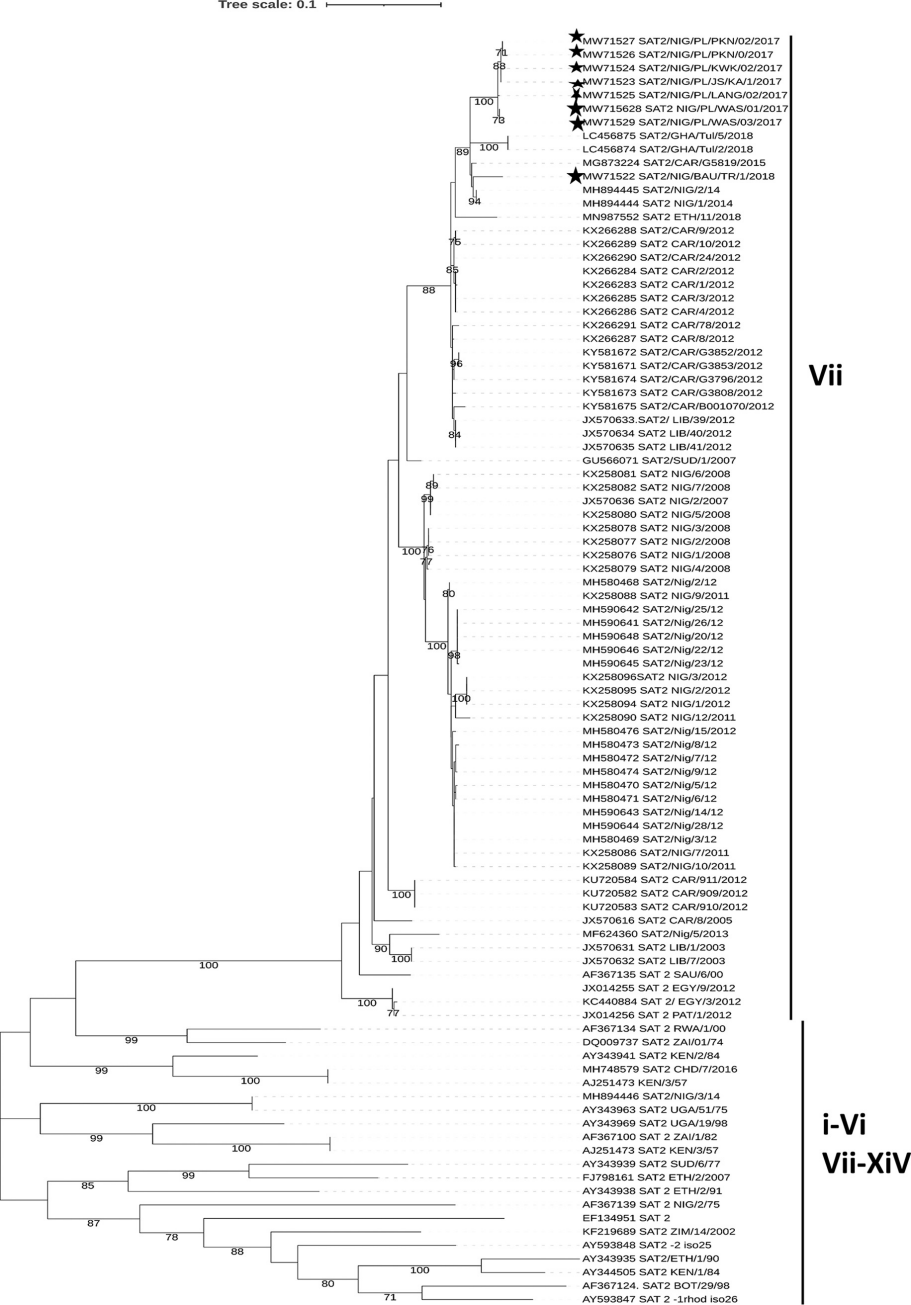
The evolutionary history was inferred by using the maximum likelihood method and the Hasegawa-Kishino-Yano model. The tree with the highest log likelihood (–9305.89) is shown. The percentage of trees in which the associated taxa clustered together is shown next to the branches. Initial trees for the heuristic search were obtained automatically by applying the neighbor-join and BioNJ algorithms to a matrix of pairwise distances estimated using the maximum composite likelihood (MCL) approach and then selecting the topology with the superior log likelihood value. A discrete gamma distribution was used to model evolutionary rate differences among sites (5 categories [+G, parameter = 0.3453]). This analysis involved 94 nucleotide sequences. All positions containing gaps and missing data were eliminated (complete deletion option). There was a total of 635 positions in the final data set. Evolutionary analyses were conducted in MEGA X. The Nigerian isolates in this study are represented by dark stars. The original tree was exported from MEGA X as a Newick tree into ITOL v5 (https://itol.embl.de) for tree display and annotation. A bootstrap value of ≥70% was considered significant. The Roman numerals i to xiv represent the 14 known topotypes of FMDV SAT2.

The VP1 sequences in this study clustered within topotype VII of SAT2 and were closely related to 2018 isolates from Ghana. The 2017 PL isolates clustered together (Fig. 1). The 2018 BAU isolate was separated from the PL isolates and was closely related to isolates from Nigeria in 2014 and Cameroon in 2015.

SAT2, previously restricted to southern Africa, has become established in other sub-Saharan African countries, with sporadic spread into North Africa and the Middle East. Topotype VII is the predominant SAT2 outside southern Africa, and our data confirm its presence in Nigeria in 2017 to 2018, in agreement with recent reports of SAT2 in Nigeria, Cameroon, and other West African countries from 2013 to 2018 (5, 19). Animal movement, especially for trade in Nigeria from neighboring Cameroon, Chad, and Sudan (6, 20), facilitates FMDV spread (21). Therefore, the FMD situation in Nigeria and neighboring countries is dynamic, and a regional control strategy remains vital.

### Data availability.

The sequences in this report and the associated raw data have been deposited in GenBank and the NCBI SRA under the accession numbers and identifiers shown in Table 1.
